# *Cotesiacassina* sp. nov. from southwestern Colombia: a new gregarious microgastrine wasp (Hymenoptera, Braconidae) reared from the pest species *Opsiphanescassina* Felder & Felder (Lepidoptera, Nymphalidae) feeding on *Elaeis* oil palm trees (Arecaceae)

**DOI:** 10.3897/zookeys.1061.67458

**Published:** 2021-09-28

**Authors:** Geraldo Salgado-Neto, Consuelo Alexandra Narváez Vásquez, Dillon S. Max, James B. Whitfield3

**Affiliations:** 1 Pós-graduação em Agronomia, Departamento de Defesa Fitossanitária, Universidade Federal de Santa Maria, 97105-900, Santa Maria, RS, Brazil Universidade Federal de Santa Maria Santa Maria Brazil; 2 Pós-graduação em Entomologia, Departamento de Entomologia/BIOAGRO, Universidade Federal de Viçosa, 36570-900, Viçosa, MG, Brazil Universidade Federal de Viçosa Viçosa Brazil; 3 Department of Entomology, 320 Morrill Hall, 505 South Goodwin Ave., University of Illinois at Urbana-Champaign, Urbana, IL 61801, USA University of Illinois Urbana United States of America

**Keywords:** Butterfly, DNA barcode, integrative taxonomy, morphology, natural enemy, new species

## Abstract

A new species of microgastrine wasp, *Cotesiacassina* Salgado-Neto, Vásquez & Whitfield, **sp. nov.**, is described from southwestern Colombia in Tumaco, Nariño. This species is a koinobiont gregarious larval endoparasitoid, and spins a common mass of cocoons underneath the host caterpillars of *Opsiphanescassina* (Felder & Felder) (Lepidoptera, Nymphalidae), feeding on oil palm trees (interspecific hybrid *Elaeisoleifera* × *E.guineensis*) (Arecaceae). While superficially similar, both morphologically and biologically, to *C.invirae* Salgado-Neto & Whitfield from southern Brazil, the two species are distinct based on DNA barcodes, host species, geographical range and morphological characters.

## Introduction

The nymphalid butterfly *Opsiphanescassina* Felder & Felder occurs from Mexico to the Amazon Basin (Brazil, Bolivia, Colombia, Ecuador, French Guiana, Guyana, Peru, Suriname and Venezuela) (Lamas 2004). This species is widespread in Colombia but is most commonly found within the States of Nariño, Cauca and Putumayo (Benavides and Cárdenas 1970; Fuentes 1973; Pava et al. 1983; Posada 1989; Villegas 1993). *Opsiphanescassina* is considered a pest of oil palm trees (interspecific hybrid *Elaeisoleifera* × *Elaeisguineensis*) (Arecaceae) in south-west Colombia (Genty 1978; Mexzón and Chinchilla 1996; Posada and Cárdenas 1996; Loría et al. 2000; González 2011). In southwestern Colombia, the occurrence of the subspecies *O.cassinanumatius* Fruhstorfer was recorded by Martínez (1970). We also recorded the presence of three additional subspecies: *O.cassinachiriquensis* Stichel, *Opsiphanescassinaperiphetes* Fruhstorfer, and *Opsiphanescassinacaliensis* Bristow.

Five species of Braconidae have been recorded as endoparasitoids of species of *Opsiphanes* (larval stage): *Cotesiabiezankoi* (Blanchard), *Cotesiaopsiphanis* (Schrottky), *Cotesiaalia* (Muesebeck) (Mason 1981; Penteado-Dias 1987; De Santis 1989; Salgado-Neto 2013), *Cotesiainvirae* Salgado-Neto & Whitfield (Salgado-Neto et al. 2019), and *Rhysipolis* sp. (Sauer 1946; Costa Lima 1950, 1962; Silva et al. 1968; Briceño-Vergara 1978; De Santis 1980; Mason 1981; Penteado-Dias 1987; Briceño-Vergara 1997; Mexzón 1997; Rodríguez et al. 2006). Here we describe a new species of *Cotesia* Cameron, reared from *O.cassina* feeding on oil palms in southwestern Colombia.

*Cotesia* is easily recognizable morphologically among microgastrine braconids, although the huge variety of species can be difficult to distinguish from each other (Whitfield et al. 2009), especially those without host data. The wasps have a koinobiont habit (Kankare and Shaw 2004) and both solitary and gregarious species are known. *Cotesia* (Braconidae, Microgastrinae) currently contains roughly 300–400 described species (Fernandez-Triana et al. 2020), but this number will certainly increase dramatically, as world estimates range from 1000–2000 species (Mason 1981; Michel-Salzat and Whitfield 2004; Whitfield et al. 2018; Fernandez-Triana et al. 2020), and a relatively small number of studies recording Neotropical species of *Cotesia* and their biology are available so far (Whitfield 1997; Whitfield et al. 2018), particularly in South America.

As *Cotesia* species appear to be highly host specialized (Kankare and Shaw 2004), with many cryptic species and geographically restricted distributions (Fiaboe et al. 2017), the use of an integrative taxonomic approach (combining morphological, molecular, biological and geographical data) is critical for recognizing and distinguishing these parasitoid wasps (Smith et al. 2008; Kaiser et al. 2017).

**Figure 1.**
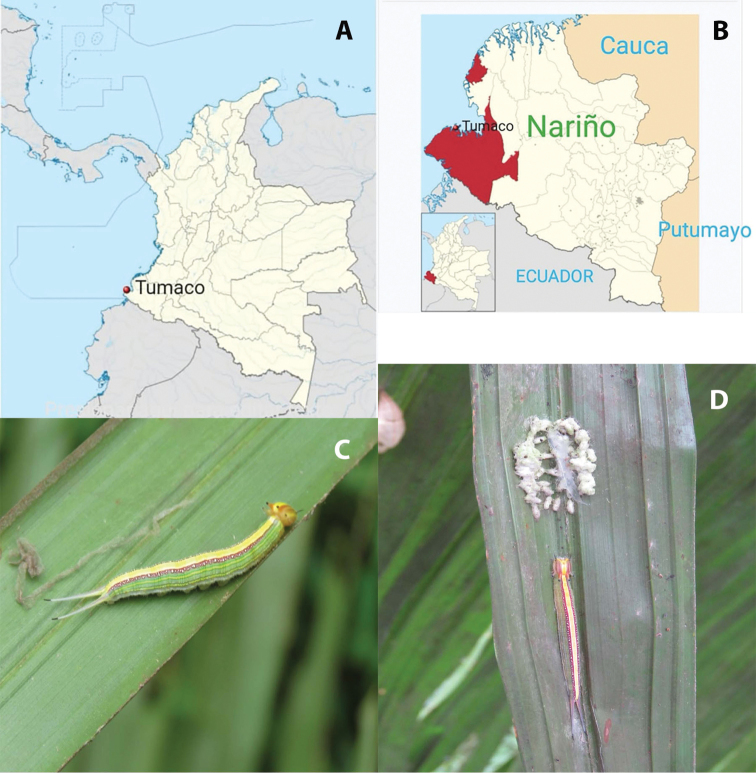
**A** simplified map of Colombia, showing rough location of Tumaco **B** close-up of southwestern Colombia, with location of Tumaco highlighted **C** caterpillar of *Opsiphanescassina* on frond of the palm *Elaeisoleifera* × *E.guineensis***D** same as **C** but with cocoons of emerged *Cotesiacassina* arranged below (normally underneath caterpillar).

Using such an integrative taxonomic approach, this paper provides a description of a new species of *Cotesia*, whose brood was produced from caterpillars of *Opsiphanescassina* (Felder & Felder) (Lepidoptera, Nymphalidae) (Fig. 1) feeding on palm trees (interspecific hybrid *Elaeisoleifera* × *E.guineensis*) (Arecaceae) in Tumaco, Nariño, south-west Colombia. We compare it with the other described species of *Opsiphanes* that have been formally recorded from the Neotropical region, two of which have been well characterized and two of which are of uncertain identity.

## Materials and methods

Between April 2018 and March 2019; we collected 35 larvae of *Opsiphanescassina* as part of a survey carried out on exotic palms in the Palmeiras plantation A.S., 58 km from San Andrés de Tumaco, Nariño, Colombia (1°47'28.0"N, 78°47'33.9"W, 28 m elev. – see Fig. 1A, B). The larvae of *O.cassina* were found on the interspecific hybrid *Elaeisoleifera* × *E.guineensis* (Arecaceae). Upon collecting, larvae were kept in the laboratory (25 ± 1 °C; 70% RH; photoperiod of 14 hours of light) and observed daily until the emergence of the butterflies or parasitoids, which were then preserved in 70% ethanol.

**Figure 2.**
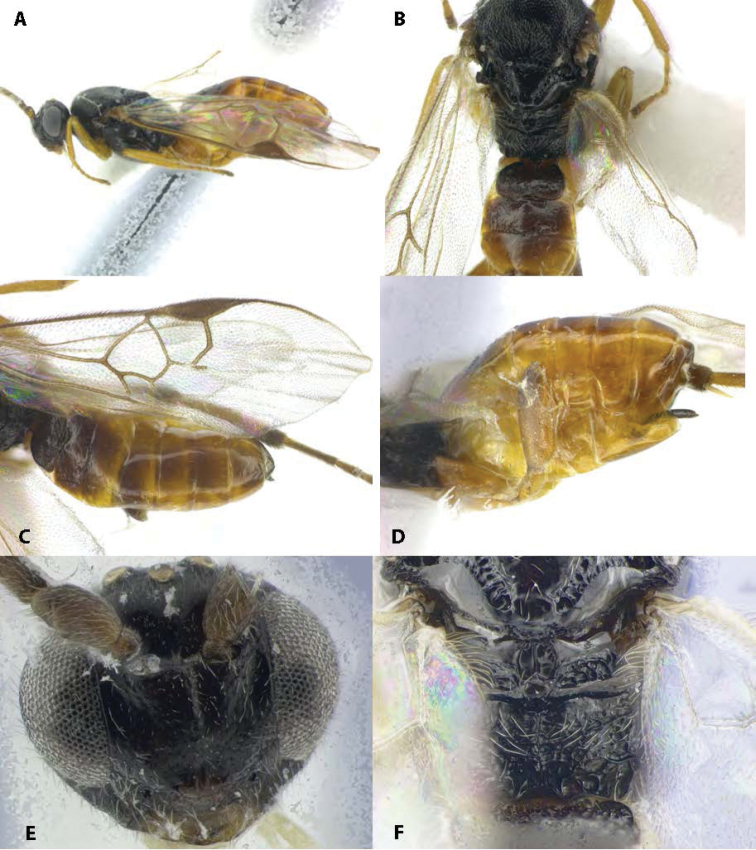
*Cotesiacassina*, sp. nov. **A** lateral habitus **B** dorsal view of mesosoma and anterior metasomal tergites **C** fore wing **D** lateral view of metasoma with hind leg removed, showing laterotergites, sternites, hypopygium and ovipositor sheaths **E** frontal view of head **F** dorsal view of posterior portions of mesosoma, especially propodeum.

Photographs of the caterpillar and parasitoid cocoons (Fig. 1C, D) were taken in the field by CANV. Morphological photographs of the *Cotesia* (Fig. 2A–F) were taken by DSM at the University of Illinois, USA using a Leica M205 C stereo microscope (467 nm resolution) fitted with a 5 megapixel Leica DFC 425 digital microscope camera. Images were stacked using a motor drive on the microscope and Zerene Stacker software. Morphological terms and measurements of structures are mostly those used by Salgado-Neto et al. (2019).

To characterize and compare the new species at the molecular level, the mitochondrial (DNA barcode) gene cytochrome oxidase I (COI) was analyzed. For the ampliﬁcation of a fragment of approximately 460 bp of this gene, we used the following primer pair: COI-F (5’-GATTTTTTGGKCAYCCMGAAG-3’) and COI-R (5’CRAATACRGCTCCTATWGATAAWAC-3’) (Gusmão et al. 2010). DNA extraction of one specimen was performed with the GenElute Mammalian Genomic DNA Miniprep Kit (Sigma-Aldrich) and followed the manufacturer’s protocol. The product was ampliﬁed via Polymerase Chain Reaction (PCR) according to the following schedule: 94 °C for 2 minutes, 40 cycles of 94 °C for 30 seconds, 54 °C for 30 seconds, 72 °C for 40 seconds and 72 °C for 4 minutes. Then the PCR product was puriﬁed using polyethylene glycol precipitation (PEG; Schmitz and Riesner 2006). These samples were sequenced using the Big Dye 3.1 reagent (Life Technologies) and a 3500 XL automatic sequencer (Life Technologies).

## Descriptive taxonomy

### Cotesia cassina

Taxon classificationAnimaliaHymenopteraBraconidae

Salgado-Neto, Vásquez & Whitfield sp. nov.

8ECBA0B1-4625-500A-B9CB-DD37DC1193F8

http://zoobank.org/3C8E3CC9-C4D2-4221-BE00-6652F17F5BD4

#### Material examined.

***Holotype*** Female, Colombia: Nariño, San Andrés de Tumaco (1°47'28.0"N, 78°47'33.9"W, 28 m elev.), March 2019, coll. Consuelo Vásquez, ex larva *Opsiphanescassina* Felder & Felder (Lepidoptera, Nymphalidae). Deposited in the collection of the National University of Colombia (UNC, Dr Fernando Fernandez, curator). ***Paratypes*** 2 males, deposited in UNC, same data as holotype. 1 female, also same data as holotype, deposited in the Illinois Natural History Survey (INHS). ***Non-types*.** 2 females, in poor condition, also deposited at INHS.

**Table 1.** Diagnostic morphological characters distinguishing *Cotesiacassina* sp. nov. from the Brazilian *C.invirae* Salgado-Neto & Whitfield.

Character	*C.invirae*	*C.cassina*
Color	Generally lighter. T3 and all tergites posterior to T3 mostly bright yellow orangish. Mesopleuron with some light yellow/brown on ventral side	Generally darker. T3 and all tergites posterior to T3 are more brown to black rather than orangish. Mesopleuron almost entirely black
T2 Sculpture	Mostly smooth. Sculpture is more uniform across width; less punctate laterally	More punctate laterally, smooth medially
T2 Shape	Posterior margin/groove straight	Posterior margin slightly convex apically, with length greatest medially

#### Diagnosis.

As discussed above, *Cotesia* is a huge worldwide genus of hundreds of species, with many morphologically similar species. While useful world identification keys are not available, it is currently possible to successfully diagnose species regionally, especially combined with molecular and host data. The closest described species, morphologically, biologically and within the region, is *Cotesiainvirae* from southern Brazil, which also parasitizes *Opsiphanes* on palms (different species). The table below provides a diagnostic comparison between the two species.

*Cotesiaalia* (Muesebeck), also recorded from *Opsiphanes*, resembles these two species but has a relatively longer first metasomal tergite (see illustration in Muesebeck 1958). Like *C.cassina*, the second tergite has the medial part of the second tergite longer than the lateral portions, and the tergites tend to both be blackish (tending to be mostly orangish in *C.invirae*). The other two named *Cotesia* species recorded from *Opsiphanes*, *C.biezankoi* (Blanchard) and *C.opsiphanis* (Schrottky), are both very poorly characterized in their descriptions and their type locations are unknown (Fernandez-Triana et al. 2020), so they are not compared here. There is a possibility that *C.invirae* might prove to be a junior synonym of *C.biezankoi*, based on shared host and geographic region, if the holotype of the latter were to resurface and be examined. Our understanding of the correct nomenclature for the entire complex would benefit from a full review of the named and putative unnamed species across all of Central and South America, especially if all the types could eventually be located. In the meantime, it is possible to characterize the relationships among the species for which we can clearly establish the identity.

#### Description.

**Female.** Body length 3.1–3.3 mm; fore wing length 2.9–3.1 mm. **Coloration** (Fig. 2A–F). General body coloration black except: scape shading from light to dark brown, palps pale yellow, tegulae brown, fore legs all yellowish, middle legs all yellowish, hind legs all yellowish except distal end of femur brown/black dorsally; distal end of tibia brown, coxae translucent yellowish, laterotergites yellowish ventrally, shading to brown dorsally; sternites and hypopygium translucent yellowish. ***Head*** (Fig. 2A, E). Facial sculpture weakly punctate; vertex sculpture smooth to very weakly punctate; distance between posterior ocellae nearly identical with distance from outer ocelli to compound eyes. ***Mesosoma*** (Fig. 2A, B, F). Pronotum with both dorsal and ventral grooves present, ventral groove crenulate. Mesoscutum fully and distinctly but shallowly punctate; scutoscutellar scrobe slightly sunken groove and formed by 8 pits. Scutellum shield-shaped to subtriangular (anteriorly straight and posteriorly rounded) and weakly convex, weakly punctate. Mesopeuron smooth and polished throughout. Propodeum generally finely rugose/punctate with indistinct longitudinal medial carina. ***Legs*** (Fig. 2A–C). Hind coxa mostly smooth with faint sculpture on dorsal face; inner hind tibial spurs slightly longer than outer. ***Wings*** (Fig. 2B, C). Fore wing hyaline with dark brownish vein pigmentation; stigma more than 2× as long as broad, without obvious pale spot at proximal end. Metacarp extending 0.60–0.70 to end of 3Rs fold along wing edge; r approximately same length as 2RS vein and meeting it at a distinct shallow angle; vannal lobe edge roughly semicircular with distal end slightly flattened; vannal lobe fringe even and dense. **Metasoma** (Fig. 2B–D, F). Tergite 1 roughly as long as broad, evenly widening from anterior margin then rounding over posterior half, mostly rugulose; tergite 2 very weakly rugulose peripherally, mostly smooth and slightly raised centrally, roughly twice as broad as long, subrectangular with posterior margin slightly longer medially than laterally. Hypopygium with angled but blunt tip, not extending past dorsal end of metasoma; ovipositor with very sparse setae at tip.

**Male.** Similar to female except with slightly narrower metasoma.

#### Molecular data.

COI barcode deposited in GenBank (MW405620). Using the identification tools in the Barcode of Life Database (Ratnasingham and Hebert 2007), *C.cassina* is closest to *C.salebrosa* (Marshall), a primarily Eurasian species attacking geometrid larvae, at a similarity level of 97.4%. Interestingly, *C.invirae* appears closest (97.02% similarity) to *Cotesia* Whitfield78 and *Cotesia* Whitfield20, two apparently conspecific sets of rearings of an undescribed species from the Lepidoptera Inventory of the Guanacaste Conservation Area (ACG) in northwest Costa Rica (Janzen et al. 2009); these rearings are from another species of *Opsiphanes*. It thus appears that there is a complex of at least four closely related species attacking different *Opsiphanes* species in a variety of geographically dispersed Neotropical habitats, as suggested by Salgado-Neto et al. (2019). BOLD and NCBI use slightly different criteria to make cutoffs in sequence comparisons, and to calculate % similarity. They also contain different sets of sequences. We checked the BOLD investigations of related species by using BLASTn (Altschul et al. 1990) to query the NCBI nucleotide database (NCBI 1988). The same most closely related species to *Cotesiacassina* and *C.invirae*, respectively, were recovered, with the exception that for *C.cassina*, *C.melitaearum* (Wilkinson) and *C.koebelei* (Riley), both attacking other genera of Nymphalidae but in the Holarctic region, joined *C.salebrosa* as closest, at roughly 94.6–95.6% similarity for all of them. In neither the BOLD nor the NCBI search did *C.cassina* come within 2.5% similarity of any other known *Cotesia* species.

#### Host.

*Opsiphanescassina* (Felder & Felder) (Lepidoptera, Nymphalidae) (Fig. 1C, D).

#### Biology/ecology.

*Cotesiacassina* is a gregarious parasitoid wasp that occurs mainly in the wet season (March-May); however, their host, *O.cassina*, occurs throughout the year, mainly in the rainy season (March-July). *Cotesiacassina* larvae kill the host larva before the end of the last instar and form their cocoons in a regular mass of dirty whitish cocoons, regularly arranged disposed under the host (Fig. 1B). The larvae of this gregarious species all emerge from the host in a short time through many different holes in the host cuticle and spin a common woolly cocoon mass within which the individual cocoons can be distinguished.

#### Distribution.

Known so far from San Andrés de Tumaco, Nariño, Colombia (Neotropical Region).

#### Etymology.

The specific epithet *cassina*, is a reference to *Opsiphanescassina* (Felder & Felder) (Lepidoptera, Nymphalidae), the host caterpillar name. The word cassina is the feminine of cassino which in Italian means playhouse.

## Supplementary Material

XML Treatment for Cotesia cassina
